# TAAR8 Mediates Increased Migrasome Formation by Cadaverine in RPE Cells

**DOI:** 10.3390/cimb46080510

**Published:** 2024-08-07

**Authors:** Joon Bum Kim, Ji-Eun Bae, Na Yeon Park, Yong Hwan Kim, Seong Hyun Kim, Hyejin Hyung, Eunbyul Yeom, Dong Kyu Choi, Kwiwan Jeong, Dong-Hyung Cho

**Affiliations:** 1 School of Life Sciences, BK21 FOUR KNU Creative BioResearch Group, Kyungpook National University, Daegu 41566, Republic of Korea; kss3213@naver.com (J.B.K.); yeonie5613@gmail.com (N.Y.P.); yoo035913@gmail.com (Y.H.K.); kgj010@naver.com (S.H.K.); itzmehyejin@gmail.com (H.H.); yeb@knu.ac.kr (E.Y.); dongkyu@knu.ac.kr (D.K.C.); 2 KNU G-LAMP Research Group, KNU Institute of Basic Sciences, College of Natural Sciences, Kyungpook National University, Daegu 41566, Republic of Korea; loveg730@naver.com; 3 Bio Industry Department, Gyeonggido Business & Science Accelerator, Suwon 16229, Republic of Korea; assylum@gbsa.or.kr; 4 Organelle Institute, Kyungpook National University, Daegu 41566, Republic of Korea

**Keywords:** migrasome, cadaverine, TAAR8, TSPAN4, RPE cells

## Abstract

Migrasomes, the newly discovered cellular organelles that form large vesicle-like structures on the retraction fibers of migrating cells, are thought to be involved in communication between neighboring cells, cellular content transfer, unwanted material shedding, and information integration. Although their formation has been described previously, the molecular mechanisms of migrasome biogenesis are largely unknown. Here, we developed a cell line that overexpresses GFP-tetraspanin4, enabling observation of migrasomes. To identify compounds that regulate migrasome activity in retinal pigment epithelial (RPE) cells, we screened a fecal chemical library and identified cadaverine, a biogenic amine, as a potent migrasome formation inducer. Compared with normal migrating cells, those treated with cadaverine had significantly more migrasomes. Putrescine, another biogenic amine, also increased migrasome formation. Trace amine-associated receptor 8 (TAAR8) depletion inhibited migrasome increase in cadaverine-treated RPE cells, and cadaverine also inhibited protein kinase A phosphorylation. In RPE cells, cadaverine triggers migrasome formation via a TAAR8-mediated protein kinase A signaling pathway.

## 1. Introduction

Migrasomes are newly discovered organelles (0.5–3 µm) with critical roles in cell migration and communication. These unique vesicular structures, which emerge as cells migrate, form on the retraction fibers pulled from the rear ends of migrating cells [1]. The retraction fibers, which extend from the cell’s trailing edge, break as the cell moves forward, leaving behind migrasomes [2], which can release cellular contents, including vesicles and the cytosol, into the extracellular environment. This indicates that migrasomes may have a significant role in extracellular signaling and material exchange between cells. Inhibiting cell migration using the myosin II inhibitor, blebbistatin, or via cell adhesion modulation also suppresses migrasome formation [3], underscoring the integral relationship between migrasome production and cell migration machinery. Tetraspanin family members, which span the cell membrane four times, are involved in various cellular processes, including cell adhesion and membrane compartmentalization [4,5]. Recently, tetraspanin family members have been identified as key migrasome formation regulators, with tetraspanin (TSPAN) 4 and TSPAN7 overexpression inducing migrasome formation. Moreover, migrasome formation is significantly impaired by knocking out the most highly expressed migrasome-forming tetraspanins [5,6]. TSPAN4, which is involved in the final migrasome formation step, is one of the most effective cell migration-inducing tetraspanins [7,8], and Liang Ma et al. have shown that green fluorescent protein (GFP)-tagged TSPAN4 can mark migrasomes [1]. Additionally, it localizes to the cell membrane and the retraction fibers [9,10]. Although migrasome formation and function involve a complex interplay between proteins and cell membrane biophysical properties, the underlying molecular mechanisms are largely unknown. However, tetraspanins are crucial in tetraspanin-enriched macrodomain formation [11,12], which creates a specialized membrane environment that facilitates migrasomes bending and budding from retraction fibers [8]. Migrasomes are reported to play a crucial role in zebrafish embryo development [1]. During zebrafish gastrulation, a critical embryonic development phase during which the basic body plan is established, migrasomes are involved in developmental cue delivery to specific embryo regions. A recent study showed that during zebrafish development, migrasomes provide regional signals that guide organ morphogenesis [6]. Previous studies indicate that migrasomes function as non-autonomous cell–cell communication organelles that influence the behavior and development of other cells [13]. Despite their roles in various physiological and pathological contexts, the mechanisms regulating migrasome formation are largely unelucidated. Here, we identified cadaverine as a novel migrasome formation activator.

Cadaverine, a linear diamine, is commonly associated with the decomposition of amino acids in biological systems. Thus, it has been implicated in various physiological processes, including cellular signaling, and plays a role in modulating cell migration and proliferation [14]. Recent studies have suggested that cadaverine interacts with the trace amine-associated receptor (TAAR) family, particularly TAAR8 [15]. However, the role of cadaverine and TAAR on migrasome has not been elucidated. This study investigates the effect of cadaverine on migrasome in RPE/GFP-TSPAN4 cells and identifies the associated signaling pathways.

## 2. Materials and Methods

### 2.1. Reagents

Cadaverine (#SML0583) and putrescine (#51799) were purchased from Sigma–Aldrich (St. Louis, MO, USA). Anti-TAAR8 (5′-CAACCUGUUGUGCAGCUUU-3′) short interfering RNA (siRNA) and scrambled siRNA (5′-CCUACGCCACCAAUUUCGU-3′) were synthesized by Genolution (Seoul, Republic of Korea).

### 2.2. Cell Culture and Image-Based Fecal Metabolite Library Screening

The GFP-tagged mouse Tspan4 plasmid was kindly provided by Dr. Li Yu (Tsinghua University, China). We generated RPE/GFP-TSPAN4 cell line stably expressing GFP-TSPAN4 in human telomerase-immortalized RPE cells. They were cultured in Dulbecco’s Modified Eagle Medium supplemented with 10% fetal bovine serum and 1% penicillin/streptomycin (Invitrogen, Carlsbad, CA, USA). For image-based screening using a fecal metabolite library (MetaSci, Toronto, ON, Canada), RPE/GFP-TSPAN4 cells were seeded in 96-well plates at a density of 3 × 10^3^ cells/well. After culturing for 24 h, the cells were treated with metabolites at 20 or 100 μM and then cultured for 24 h before examination under a fluorescence microscope (IX71, Olympus, Tokyo, Japan) for activated migrasome detection.

### 2.3. Migrasome Counting and Live Cell Imaging

Migrasomes were counted under a fluorescence microscope in about 200 cells per experimental condition (*n* = 3), and the percentage of migrasome-containing cells was determined using the following formula: (total number of migrasome-containing cells/total number of nuclei per image) × 100. Using the CellSense Standards software 4.2 CS-ST-V4.2 (Olympus Europa Holding GmbH, Hamburg, Germany), the migrasome diameter was determined with the free-hand line selection tool. Average migrasome numbers per cell were calculated. GraphPad Prism 8 (GraphPad Software, San Diego, CA, USA) was used for graphical data analysis. Time-lapse live cell imaging was conducted on an Operetta CLS system at 37 °C and 5% CO_2_, using a 20× high NA objective lens (NA 0.8) in widefield mode. Images were acquired for 24 h. After RPE/GFP-TSPAN4 cell treatment with cadaverine and putrescine, initially, the time series was started at short measurement 30-min intervals.

### 2.4. Western Blot Analysis

Cells were lysed using 2× Laemmli sample buffer (1610737, Bio-Rad, Hercules, CA, USA), and protein concentration was quantified using the Bradford dye method (5000001, Bio-Rad) as per the manufacturer’s protocol. Proteins were then resolved using SDS polyacrylamide gel electrophoresis, transferred onto PVDF membranes (1620177, Bio-Rad), blocked using 4% skimmed milk (BD Bioscience, San Diego, CA, USA, 90002–594) in TBST (Tris base [T9200, GenDEPOT, Baker, TX, USA], NaCl [G0610, GenDEPOT], Tween^®^ 20 [P7949] Sigma-Aldrich). Next, the membrane was incubated with primary antibodies against TAAR8 (BS-12023R, Thermo Fisher Scientific, Waltham, MA, USA), p-protein kinase A (PKA) (4781S, Cell Signaling Technology, Danvers, MA, USA), PKA (4782S, Cell Signaling Technology) and ACTA1 (MAB1501, Sigma–Aldrich). They were then incubated with HRP-conjugated secondary antibodies (7076S and 7074S, Cell Signaling Technology).

### 2.5. Statistical Analyses

Data from at least three independent experiments are presented as means ± standard deviation (SD). Differences between groups were compared using a Student’s *t*-test and one-way ANOVA, followed by a Bonferroni post hoc test. Significance levels were set at ** *p* < 0.01 and *** *p* < 0.001.

## 3. Results and Discussion

### 3.1. Cadaverine and Putrescine Promotes Migrasome in RPE/GFP-TSPAN4 Cells

**Figure 1:**
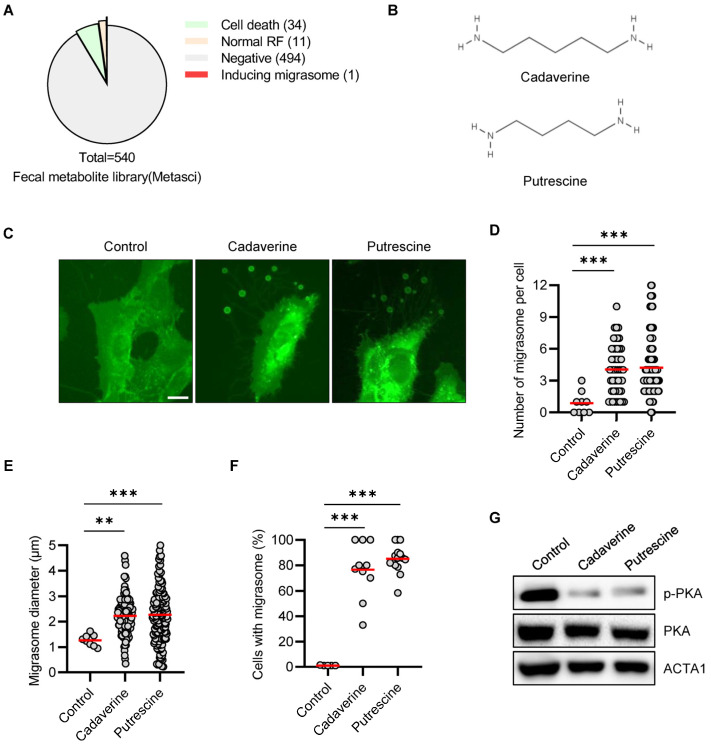
Cadaverine and putrescine promote migrasome formation in RPE/GFP-TSPAN4 cells (**A**) A pie chart of the number of compounds in each phenotypic category. (**B**) The chemical structures of the diamine compounds, cadaverine and putrescine. (**C**) RPE/GFP-TSPAN4 cells were treated with cadaverine (50 μM) or putrescine (50 μM) for 24 h before being imaged under a fluorescence microscope. (**D**–**F**) Cadaverine- and putrescine-treated (24 h) RPE/GFP-TSPAN4 cells were counted migrasome numbers per cell (**D**), migrasome diameter, (**E**), and the number of migrasome-containing cells were counted (**F**). (**G**) Western blot analysis using the indicated antibodies. Measurements were made in about 200 cells per group. The red line represents the average value of the data. Data are presented as means ± SD. Statistical significance was assessed using one-way ANOVA, with ** and *** indicating *p* < 0.01 and <0.001, respectively. Scale bar: 20 μm.

Migrasomes are recently discovered organelles generated during cell migration. To identify migrasome formation-inducing molecules and their target proteins, we first screened a fecal metabolite library containing 540 metabolites using RPE cells stably expressing GFP-TSPAN4 (RPE/GFP-TSPAN4) to monitor migrasomes (Table S1). This analysis identified only one putative target that significantly induced migrasome generation (Figure 1A). Cadaverine (pentane-1,5-diamine/NH_2_(CH_2_)_5_NH_2_) is a linear diamine chemical, while putrescine (butane-1,4-diamine/NH_2_(CH_2_)_4_NH_2_) is also classified as a linear diamine with a similar chemical formula to cadaverine, but with one fewer hydrocarbon chain (Figure 1B). To explore their relationship, we examined if linear diamine enhances migrasome formation. This analysis showed that treating RPE/GFP-TSPAN4 cells with cadaverine or putrescine markedly increased migrasome formation (Figure 1C and Figure S1). Furthermore, compared with control cells, migrasome numbers per cell, migrasome diameter, and the number of migrasome-containing cells increased significantly (Figure 1D–F). The video in Movie S1 shows migrasome generation during RPE/GFP-TSPAN4 cell movement after treatment with cadaverine. These results indicate that linear diamines may induce migrasome formation.

### 3.2. Cadaverine and Putrescine Inhibit PKA Phosphorylation in RPE Cells

Cadaverine is a ligand of the TAAR family of G protein-coupled receptors (GPCR) [15]. One key pathway involves PKA signaling cascade activation. Therefore, we investigated the effect of TAAR’s downstream signaling pathways on migrasome formation and observed that cadaverine significantly inhibited PKA activation (Figure 1G). Moreover, compared with the control group, phosphorylated PKA levels decreased in cells treated with cadaverine or putrescine.

### 3.3. TAAR8 Knock-Down Inhibits Cadaverine-Inducing Migrasome Formation

**Figure 2:**
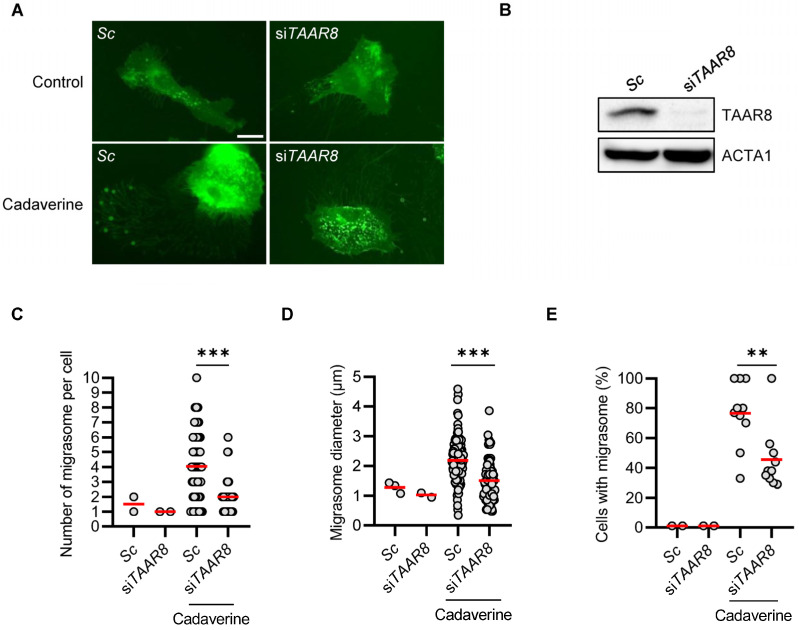
TAAR8 knockdown inhibits migrasome formation in cadaverine-treated conditions (**A**,**B**). RPE/GFP-TSPAN4 cells were transiently transfected with scrambled siRNA (Sc, negative control) or anti-TAAR8 siRNA (siTAAR8), followed by treatment with cadaverine (50 μM) for 24 h and imaging under a fluorescence microscope (**A**). TAAR8 silencing was examined using western blot analysis with the indicated antibodies (**B**). (**C**–**E**) RPE/GFP-TSPAN4 cells were transiently transfected with scrambled siRNA (Sc, negative control) or anti-TAAR8 siRNA (siTAAR8) and then treated with cadaverine (50 μM) for 24 h. They were then fixed, and migrasome numbers per cell (**C**), migrasome diameter (**D**), and the number of migrasome-containing cells were then determined (**E**). Measurements were made in about 200 cells per group. The red line represents the average of the data. Data are presented as means ± SD. Statistical significance was assessed using one-way ANOVA, with ** and *** indicating *p* < 0.01 and <0.001, respectively. Scale bar: 10 μm.

Cadaverine and putrescine bind to TAAR8 and are involved in various physiological processes, including cell migration [15]. Since our findings indicated that cadaverine and putrescine enhance migrasome formation, we investigated TAAR8’s role in migrasome formation further in cadaverine-treated cells and, notably, observed that TAAR8 inhibited the cadaverine-induced increase in migrasomes in RPE/GFP-TSPAN4 cells (Figure 2A,B). Consistently, compared with cadaverine-treated cells, migrasomes number per cell, migrasome diameter, and the percentage of migrasome-containing cells decreased in TAAR8-silenced cells (Figure 2C–E), suggesting that TAAR8 controls migrasome formation in cadaverine-treated cells. Our study provides a valuable tool for identifying and validating chemicals and their target proteins involved in migrasome formation mechanisms. From the screening conducted in this research, we identified linear diamines, cadaverine, and putrescine, which induced migrasome biogenesis with significant fiber retraction (Figure 1). Additionally, TAAR8 was identified as a key regulator of migrasome formation, serving as the target protein for cadaverine (Figure 2). 

TAAR superfamily proteins, which belong to the GPCR family, are characterized by a common structural framework that includes seven transmembrane helices, an extracellular N-terminus, and an intracellular C-terminus. This configuration allows them to function as cellular receptors that transduce external signals into cellular responses. Functionally, TAARs serve as chemosensors in various physiological processes, responding to amines and other ligands [16]. Upon binding their specific ligands, TAARs initiate a signal transduction cascade through the activation or inhibition of associated G proteins, which regulate downstream pathways such as cyclic AMP (cAMP) production. Specifically, TAAR8 is linked with inhibitory G protein (Gi) pathways, which typically reduce cellular levels of cAMP upon activation [17]. When activated by its ligand, TAAR8 inhibits adenylate cyclase through its associated Gi protein, thereby reducing cAMP production from ATP. This reduction in cAMP levels consequently downregulates PKA activity, influencing cellular functions such as migration [18]. PKA can phosphorylate and inactivate Rac1 GTPase, a key regulator of actin cytoskeleton dynamics in cell migration. The inhibition of Rac1 leads to decreased actin polymerization at the cell front, thus reducing cell motility [19]. Conversely, PKA can also act as a positive regulator by phosphorylating targets involved in cytoskeletal dynamics, such as LIM kinase, which in turn phosphorylates and inactivates cofilin, an actin-depolymerizing factor [20]. This action stabilizes actin filaments and facilitates lamellipodia formation at the leading edge of migrating cells, enhancing motility. Therefore, the function of PKA in cell migration is highly context-dependent, influenced by factors such as cell type, the cellular environment, and the specific set of signaling molecules expressed. In our study, we found that treatment with cadaverine as a TAAR8 ligand inhibits PKA phosphorylation in RPE cells (Figure 1G). Furthermore, the enhanced migrasome formation by cadaverine is reduced through TAAR8 depletion (Figure 2), suggesting that Gi signaling with cAMP-PKA regulates migrasomes in cadaverine-treated cells. This study highlights the intricate molecular mechanisms by which TAAR8 and cAMP-PKA signaling pathways influence migrasome formation. These findings provide a foundation for future research into the physiological and pathological roles of migrasomes, potentially leading to novel controlling strategies linked to altered migrasome dynamics.

## 4. Conclusions

In this study, we observed the effects of cadaverine and putrescine on migrasome formation in RPE/GFP-TSPAN4 cells. Our results indicate that these linear diamines significantly enhance migrasome generation. This enhancement is linked to the inhibition of PKA phosphorylation through the TAAR8 receptor, which is crucial for mediating the effects of cadaverine. Furthermore, the reduction in migrasome formation observed in TAAR8-silenced cells emphasizes the importance of TAAR8 in regulating this process. Our findings provide insights into the molecular mechanisms of migrasome dynamics and highlight the potential of targeting TAAR8 and the cAMP-PKA pathway for future research into cell migration and its related physiological and pathological roles.

## Data Availability

The data presented in this study are available on request from the corresponding author. The data are not publicly available for privacy reasons.

## Supplementary Materials

The following supporting information can be downloaded at: https://www.mdpi.com/article/10.3390/cimb46080510/s1. Table S1: List of fecal metabolites library used for migrasome screening; Figure S1: Another image corresponding to Figure 1C; Movie S1: Another image corresponding to Figure 1C.
