# Supplementary material for: TAAR8 Mediates Increased Migrasome Formation by Cadaverine in RPE Cells
Source: Curr Issues Mol Biol. 2024 Aug 7;46(8):8658–64. doi: 10.3390/cimb46080510 (PMC11352285; doi:10.3390/cimb46080510)
Supplement: Supplementary file 1 [file cimb-46-00510-s001.zip › Supplementary information.pdf]

Manuscript Type: Supplementary Information

TAAR8 mediates increased migrasome formation by Cadaverine in RPE cells

Joon Bum Kim [1], Ji-Eun Bae [2], Na Yeon Park [1], Yong Hwan Kim [1], Seong Hyun Kim [1], Hyejin Hyung [1], Eunbyul Yeom [1], Dong Kyu Choi [1], Kwiwan Jeong [3], and Dong-Hyung Cho [1,4,*]

[1] School of Life Sciences, BK21 FOUR KNU Creative BioResearch Group, Kyungpook National University, Daegu 41566, Republic of Korea
[2] KNU G-LAMP Research group, KNU Institute of Basic Sciences, College of Natural Sciences, Kyungpook National University, Daegu 41566, Republic of Korea
[3] Bio Industry Department, Gyeonggido Business & Science Accelerator, Suwon 16229, Republic of Korea.
[4] Organelle Institute, Kyungpook National University, Daegu 41566, Republic of Korea
* Correspondence: dhcho@knu.ac.kr; Tel.: +82-53-950-5382

SUPPLEMENTARY INFORMATION

Supplementary Table S1. List of fecal metabolites library used for migrasome screening

Table S1. List of fecal metabolites library used for migrasome screening.

No.	Name	CAS number
1	Betaine	107-43-7
2	L-Glutamine	56-85-9
3	Glutaconic acid	1724-02-3
4	Sarcosine	107-97-1
5	Fumaric acid	110-17-8
6	Indole-3-acetic acid sodium salt	6505-45-9
7	L-Tyrosine	60-18-4
8	Mandelic acid	90-64-2
9	Creatine monohydrate	6020-87-7
10	L-Carnitine inner salt	541-15-1
11	3-Aminoisobutanoic acid hydrate	144-90-1
12	Ferulic acid	1135-24-6
13	Taurine	107-35-7
14	4-Hydroxybenzoic acid	99-96-7
15	Inosine	58-63-9
16	Pyridoxine	65-23-6
17	Urea	57-13-6
18	Ascorbic acid	50-81-7
19	Adenosine	58-61-7
20	L-Glutamic acid	56-86-0
21	Folic acid	59-30-3
22	Myoinositol	87-89-8
23	Creatinine	60-27-5
24	Tyramine	51-67-2
25	Pimelic acid	111-16-0
26	D-(+)-Glyceric acid Hemicalcium salt	207300-72-9
27	L-Leucine	61-90-5
28	Thymine	65-71-4
29	Glyoxylic acid monohydrate	298-12-4
30	Suberic acid	505-48-6
31	Undecanoic acid	112-37-8

32	Maleic acid	110-16-7
33	Cholic acid	81-25-4
34	Uracil	66-22-8
35	Tetradecanedioic acid	821-38-5
36	Xanthosine	146-80-5
37	Uric acid	69-93-2
38	Deoxycholic acid	83-44-3
39	D-α-aminobutyric acid	2623-91-8
40	Uridine	58-96-8
41	Galactitol	608-66-2
42	Xanthine	69-89-6
43	Allantoin	97-59-6
44	L-Serine	56-45-1
45	3-Methylindole	83-34-1
46	L-Phenylalanine	63-91-2
47	Tridecanoic acid	638-53-9
48	D-(+)-Xylose	58-86-6
49	Niacinamide	98-92-0
50	2-Oxo-3-phenylpropanoic acid	156-06-9
51	Malonic acid	141-82-2
52	Ribitol	488-81-3
53	Kynurenic acid	492-27-3
54	Stigmasterol	83-48-7
55	Hexadecanoic acid	57-10-3
56	L-Proline	147-85-3
57	L-Homoserine	672-15-1
58	L-Asparagine	70-47-3
59	L-Aspartic acid	56-84-8
60	L-Glutathione reduced	70-18-8
61	Urocanic acid	104-98-3
62	Riboflavin	83-88-5
63	Glycolic acid	79-14-1
64	α-Lactose	63-42-3
65	L-Histidine monohydrochloride monohydrate	5934-29-2
66	Sebacic acid	111-20-6
67	L-Anserine nitrate salt	10030-52-1
68	2-Furoic acid	88-14-2
69	Gentisic acid	490-79-9

70	Glutaric acid	110-94-1
71	Indole	120-72-9
72	L-Alanine	56-41-7
73	Azelaic acid	123-99-9
74	Sorbitol	50-70-4
75	Levulinic acid	123-76-2
76	Sucrose	57-50-1
77	Decanoic acid	334-48-5
78	Succinic acid	110-15-6
79	Glycine	56-40-6
80	L-Threonine	72-19-5
81	L-Tryptophan	73-22-3
82	Cholesterol	57-88-5
83	Citric acid	77-92-9
84	Guanine	73-40-5
85	δ-Gluconolactone	90-80-2
86	D-(+)-Galactose	59-23-4
87	Cytidine 5'-monophosphate	63-37-6
88	N-Acetyl-L-alanine	97-69-8
89	Orotic acid	65-86-1
90	Oxoglutaric acid	328-50-7
91	γ-Aminobutyric acid	56-12-2
92	Melibiose	585-99-9
93	Glycocholic acid hydrate	475-31-0
94	Cytosine	71-30-7
95	L-Isoleucine	73-32-5
96	Choline chloride	67-48-1
97	L-Gulonic acid γ-lactone	1128-23-0
98	Hypoxanthine	68-94-0
99	2'-Deoxyuridine	951-78-0
100	Aminoadipic acid	542-32-5
101	Octadecanoic acid	57-11-4
102	DL-5-Hydroxylysine hydrochloride	13204-98-3
103	trans-Cinnamic acid	140-10-3
104	Cholestanol	80-97-7
105	Myristic acid	544-63-8
106	Ureidosuccinic acid	923-37-5
107	Adipic acid	124-04-9

108	N-Acetylglycine	543-24-8
109	Bilirubin	635-65-4
110	Hippuric acid	495-69-2
111	1,5-Diaminopentane dihydrochloride	1476-39-7
112	1,7-dimethylxanthine	611-59-6
113	L-(−)-3-Phenyllactic acid	20312-36-1
114	(+)-Pantothenic acid, sodium salt	867-81-2
115	N-Acetylglucosamine	7512-17-6
116	2′-Deoxyguanosine	961-07-9
117	NADP hydrate	53-59-8 (anhydrous)
118	2'-Deoxycytidine	951-77-9
119	2′-Deoxyadenosine monohydrate	958-09-8
120	NADH, disodium salt hydrate	606-68-8
121	Adenosine 5'-triphosphate disodium salt	987-65-5
122	Histamine dihydrochloride	51-45-6
123	Glyceraldehyde	56-82-6
124	Cholesteryl oleate	303-43-5
125	3-Methyl-L-histidine	368-16-1
126	Deoxyadenosine monophosphate	653-63-4
127	L-allo-Isoleucine	1509-34-8
128	NAD	53-84-9
129	Dopamine hydrochloride	62-31-7
130	L-Arginine	74-79-3
131	L-Lysine	56-87-1
132	Adenosine-5'-monophosphate monohydrat	18422-05-4
133	Ergosterol	57-87-4
134	L-Homocitrulline	1190-49-4
135	L-Cysteine	52-90-4
136	L-Valine	72-18-4
137	Tricosanoic acid	2433-96-7
138	DL-4-Hydroxy-3-methoxymandelic acid	55-10-7
139	Pyridoxamine dihydrochloride	524-36-7
140	Acetamide	60-35-5
141	Guanosine 5'-monophosphate (disodium salt)	1333479
142	Oxalacetic acid	328-42-7
143	Ortho-Hydroxyphenylacetic acid	614-75-5
144	D-(-)-Fructose	57-48-7
145	Mannitol	69-65-8

146	Phosphoserine	407-41-0
147	2-Methylmalonic acid	516-05-2
148	L-Fucose	2438-80-4
149	L-Arabitol	7643-75-6
150	Biotin	58-85-5
151	N-Acetylneuraminic acid	131-48-6
152	Dodecanoic acid	143-07-7
153	3-Hydroxyphenylacetic acid	621-37-4
154	2-Hydroxycaproic acid	6064-63-7
155	L-Arabinose	5328-37-0
156	Citraconic acid	498-23-7
157	Hydrocinnamic acid	501-52-0
158	(S)-(−)-2-Hydroxyisocaproic acid	13748-90-8
159	cis-Aconitic acid	585-84-2
160	β-Glycerophosphoric acid, disodium salt pentahydrate	13408-09-8
161	3,4-Dihydroxyhydrocinnamic acid	1078-61-1
162	2-Hydroxy-3-methylbutyric acid	4026-18-0
163	D-Glucosamine hydrochloride	3416-24-8
164	D-Glucuronic acid	1700908
165	N-Acetyl-L-aspartic acid	997-55-7
166	(-)-Epicatechin	490-46-0
167	2-Hydroxyoctanoic acid	617-73-2
168	Ribose	50-69-1
169	Tartaric acid	87-69-4
170	trans-3-Hydroxycinnamic acid	588-30-7
171	DL-Isocitric acid trisodium salt hydrate	1637-73-6
172	Taurodeoxycholic acid sodium salt hydrate	516-50-7
173	Phosphorylcholine chloride calcium salt tetrahydrate	72556-74-2
174	Traumatic acid	6402-36-4
175	Ethylmalonic acid	601-75-2
176	Caffeine	58-08-2
177	Benzoic acid	65-85-0
178	2-Hydroxybutyric acid	600-15-7
179	Hydrochlorothiazide	58-93-5
180	D-Saccharic acid monopotassium salt	576-42-1
181	Ornithine HCl	3184-13-2
182	Syringic acid	530-57-4
183	4-Nitrophenol	100-02-7

184	Caffeic acid	331-39-5
185	Maltotriose	1109-28-0
186	Desaminotyrosine	501-97-3
187	Sodium cyanate	71000-82-3
188	Hydroxylamine hydrochloride	1304222
189	Adenosine 5′-diphosphate sodium salt	20398-34-9
190	Hydroquinone	123-31-9
191	p-Cresol	106-44-5
192	Ursodeoxycholic acid	128-13-2
193	Oxalic acid	144-62-7
194	Imidazole	288-32-4
195	meso-Erythritol	149-32-6
196	Sodium Thiocyanate	540-72-7
197	Diethanolamine	111-42-2
198	Quercetin	117-39-5
199	1-Methyluric acid	708-79-2
200	Gallic acid	149-91-7
201	L-Carnosine	305-84-0
202	3,4-Dimethoxyphenylacetic acid	93-40-3
203	n-Butyric acid, Na salt	156-54-7
204	Adenine hydrochloride	73-24-5
205	Tauroursodeoxycholic acid, Na salt	14605-22-2
206	Nicotinic acid	59-67-6
207	p-Aminobenzoic acid	150-13-0
208	Folinic acid calcium salt hydrate	1492-18-8
209	Theophylline	58-55-9
210	Serotonin	153-98-0
211	Guanosine	118-00-3
212	1-Methylnicotinamide chloride	1005-24-9
213	Vanillic acid	204-466-8
214	Trimethylamine N-oxide dihydrate	62637-93-8
215	L-Citrulline	372-75-8
216	L-Kynurenine	2922-83-0
217	N-Acetylglutamic acid	1188-37-0
218	Spermine tetrahydrochloride	306-67-2
219	Deoxyinosine	890-38-0
220	Cytidine 5′-triphosphate (disodium salt)	36051-68-0
221	(±)-3-Methyl-2-oxovaleric acid sodium salt	3715-31-9

222	L-Cysteic acid monohydrate	23537-25-9
223	Hexadecanedioic acid	505-54-4
224	Thymidine	50-89-5
225	Cytidine	65-46-3
226	L-Histidinol Dihydrochloride	1596-64-1
227	Sodium D-gluconate	527-07-1
228	n-Glycerol 3-Phosphate Bis(cyclohexylammonium) Salt	29849-82-9
229	1,2-Dipalmitoyl-sn-glycero-3-phosphate monosodium salt	169051-60-9
230	2-Isopropylmalic acid	3237-44-3
231	L-Hydroorotic acid	5988-19-2
232	Maltose Monohydrate	6963-53-7
233	Ethanolamine hydrochloride	2002-24-6
234	Thiamine hydrochloride	67-03-8
235	Glycodeoxycholic acid sodium salt	16409-34-0
236	D-Mannose	3458-28-4
237	Chitin	1398-61-4
238	Chenodeoxycholic acid sodium salt	2646-38-0
239	1,6-anhydro-b-D-Glucose	498-07-7
240	Xylitol	87-99-0
241	Trimethylamine hydrochloride	593-81-7
242	D-Glucose 6-phosphate sodium salt	54010-71-8
243	5-Hydroxymethyl-2-furancarboxylic acid	6338-41-6
244	Aminomalonic acid	1068-84-4
245	2-hydroxy-3-methylpentanoic acid	86540-81-0
246	All trans-Retinal	116-31-4
247	D-(+)-Trehalose dihydrate	6138-23-4
248	4-Acetamidophenol	103-90-2
249	Nonanoic acid	112-05-0
250	3-Hydroxy-3-methylglutaric acid	503-49-1
251	7-Methylxanthine	552-62-5
252	L-Pyroglutamic acid	98-79-3
253	N-α-Acetyl-L-glutamine	2490-97-3
254	Acesulfame Pottasium	55589-62-3
255	2-Methylglutaric acid	617-62-9
256	Vanillin	121-33-5
257	L-Cystine	56-89-3
258	3-Methylxanthine	1076-22-8
259	L-(−)-Malic acid	97-67-6

260	Menadione	58-27-5
261	(±)-Menthol	1490-04-6
262	trans-Aconitic acid	4023-65-8
263	N-Acetyl-L-phenylalanine	2018-61-3
264	DL-p-Hydroxyphenyllactic acid	6482-98-0
265	Phosphonoacetic acid	4408-78-0
266	D-(+)-Raffinose pentahydrate	17629-30-0
267	3,4-Dihydroxybenzeneacetic acid	102-32-9
268	Pyrogallol	87-66-1
269	Nervonic acid	506-37-6
270	Sinapic acid	530-59-6
271	D-Arabitol	488-82-4
272	1-Octadecanol	112-92-5
273	Cyanocobalamin	68-19-9
274	5β-Cholestan-3α-ol	516-92-7
275	1,3-Diaminopropane	109-76-2
276	Glycochenodeoxycholic acid sodium salt	16564-43-5
277	2-Methyllactic acid	594-61-6
278	Catechol	120-80-9
279	3-hydroxybutyric acid	300-85-6
280	Purine	120-73-0
281	1,11-Undecanedicarboxylic acid	505-52-2
282	D-(+)-Tryptophan	153-94-6
283	4-Hydroxybenzaldehyde	123-08-0
284	1-Hexadecanol	36653-82-4
285	Heptadecanoic acid	506-12-7
286	Furosemide	54-31-9
287	trans-4-Hydroxycinnamic acid	501-98-4
288	Curcumin	458-37-7
289	Isethionic acid sodium salt	1562-00-1
290	L-Rhamnose Monohydrate	10030-85-0
291	L-Methionine	63-68-3
292	Lanosterol	79-63-0
293	1-Methyl-hydantoin	616-04-6
294	Ammonium formate	540-69-2
295	4-Hydroxyphenylacetic acid	156-38-7
296	5-Hydroxyindole-3-acetic acid	54-16-0
297	DL-Threitol	7493-90-5

298	(R)-(-) Citramalic acid lithium salt	1583974
299	L-Hydroxyproline	51-35-4
300	(R)-(+)-2-Pyrrolidone-5-carboxylic acid	4042-36-8
301	Xanthurenic acid	59-00-7
302	3,5-Dihydroxybenzoic acid	99-10-5
303	Dimethylamine hydrochloride	506-59-2
304	Glycyl-glycine	556-50-3
305	Glycyl-L-tyrosine hydrate	312693-80-4
306	DL-Indole-3-lactic acid	832-97-3
307	4-Hydroxyphenylpyruvic acid	156-39-8
308	m-Hydroxyhippuric Acid	1637-75-8
309	3-Methylglutaconic Acid	5746-90-7
310	o-Toluic acid	118-90-1
311	5-Aminosalicylic acid	89-57-6
312	1-Methyl Adenosine	15763-06-1
313	3-Methylglutaric acid	626-51-7
314	Sodium L-lactate	867-56-1
315	D-Allose	2595-97-3
316	L-Norleucine	327-57-1
317	(±)-Naringenin	67604-48-2
318	Cystathionine	535-34-2
319	D-Ribose 5-phosphate disodium salt hydrate	18265-46-8
320	L-Glutathione oxidized	27025-41-8
321	Dodecanedioic acid diammonium salt	72447-43-9
322	Indole-3-carboxylic acid	771-50-6
323	Phenol	108-95-2
324	4-Hydroxy-3-methoxyphenylacetic Acid	306-08-1
325	Triamterene	396-01-0
326	Atenolol	29122-68-7
327	Theobromine	83-67-0
328	Acetoacetic acid Lithium Salt	578486
329	N-Propionylglycine	21709-90-0
330	2-[(4-Hydroxyphenyl)formamido]acetic acid	2482-25-9
331	Diltiazem HCl	33286-22-5
332	Metformin HCl	1115-70-4
333	Sodium 2-(hydroxymethyl)butanoate	1909327-68-9
334	Phenyl-Ac-Gln-OH	28047-15-6
335	O-Acetyl-L-carnitine HCl	5080-50-2

336	2-Methylsuccinic acid	498-21-5
337	1,4-Diaminobutane dihydrochloride	110-60-1
338	scyllo-Inositol	488-59-5
339	3-Hydroxymandelic acid	17119-15-2
340	Phospho(enol)pyruvic Acid Monopotassium Salt	4265-07-0
341	4-Pyridoxic acid	82-82-6
342	UDP Glucose (disodium salt)	28053-08-9
343	N-Iso valerylglycine	16284-60-9
344	5,6-Dihydro Thymine	696-04-8
345	UPD-α-D-Galactose	137868-52-1
346	Arachidic acid	506-30-9
347	2,3-Pyridinedicarboxylic acid	89-00-9
348	3-Hydroxypropionic Acid Sodium Salt	6487-38-3
349	Tetracosanoic acid	557-59-5
350	Inosine 5′-triphosphate trisodium salt	35908-31-7
351	Pyridoxal 5′-phosphate hydrate	853645-22-4
352	Elaidic Acid	112-79-8
353	L-Threonic acid Calcium Salt	70753-61-6
354	D-alanine	338-69-2
355	Dihydrouracil	504-07-4
356	L(-)-Pipecolinic acid	3105-95- 1
357	Agmatine sulfate	2482-00-0
358	D-Galactonic acid hemicalcium salt	6622-52-2
359	6-Hydroxynicotinic acid	5006-66-6
360	Lithocholic acid	434-13-9
361	N,N-Dimethylglycine hydrochloride	1118-68-9
362	Guanidoacetic acid	352-97-6
363	L-Homoarginine Hydrochloride	1483 -01-8
364	Tricarballylic acid	99-14-9
365	N-Acetyl-L-cysteine	616-91-1
366	L-α-amino-n-Butyric acid	1492-24-6
367	Saccharin sodium salt hydrate	82385-42-0
368	D-3-Phenyllactic acid	7326-19-4
369	3-Methyladipic acid	422955
370	2-Oxoadipic acid	3184-35-8
371	DL-α-Hydroxyglutaric acid disodium salt	40951-21-1
372	N-α-Acetyl-L-lysine	1946-82-3
373	Pentadecanoic acid	1002-84-2

374	Mucic acid	526-99-8
375	4-Hydroxymandelic acid monohydrate	1198-84-1
376	3,4-Dihydroxybenzoic acid	99-50-3
377	β-Alanine	107-95-9
378	Guanosine 5′-triphosphate sodium salt hydrate	36051-31-7
379	Erucic acid	112-86-7
380	Coenzyme Q10	303-98-0
381	Thiamine pyrophosphate hydrochloride	154-87-0
382	Retinol	68-26-8
383	D-Glutamic acid	6893-26-1
384	Phthalic acid	88-99-3
385	Thiamine monophosphate chloride dihydrate	273724-21-3
386	8-Aminooctanoic acid	1002-57-9
387	2-Aminoisobutyric acid	62-57-7
388	D-Glucosamine 6-phosphate	3616-42-0
389	Cortexolone	152-58-9
390	Riboflavin 5'-Monophosphate Sodium Salt	130-40-5
391	Flavin Adenine Dinucleotide Disodium Salt Hydrate	84366-81-4
392	S-(5'-Adenosyl)-L-methionine p-toluenesulfonate salt	52248-03-0
393	2-Deoxy-D-ribose	533-67-5
394	α-D-Glucose 1-phosphate disodium salt hydrate	56401-20-8
395	Apigenin	520-36-5
396	Sucralose	56038-13-2
397	Uridine-5'-phosphoric acid disodium salt	3387-36-8
398	Daidzein	486-66-8
399	Uridine 5'-diphosphate disodium salt hydrate	27821-45-0
400	N-Acetyl-D-galactosamine	1811-31-0
401	Hesperidin	520-26-3
402	5-Methyluridine	1463-10-1
403	5-Methylcytosine	554-01-8
404	DL-6,8-Thioctamide	940-69-2
405	2,6-Diaminopimelic Acid	583-93-7
406	Nonadecanoic acid	646-30-0
407	trans-4-Hydroxycyclohexanecarboxylic Acid	3685-26-5
408	Allantoic acid	99-16-1
409	5-Aminovaleric acid	660-88-8
410	Glycoursodeoxycholic acid	64480-66-6
411	Luteolin	491-70-3

412	Genistein	446-72-0
413	5-Keto-D-gluconic acid potassium salt	91446-96-7
414	D-(−)-Arabinose	10323-20-3
415	α-D-Glucose anhydrous	492-62-6
416	N-Formyl-L-methionine	4289-98-9
417	N-Acetyl-L-methionine	65-82-7
418	3-Hydroxybenzoic acid	99-06-9
419	Pyridoxal hydrochloride	65-22-5
420	trans-3-Indoleacrylic acid	29953-71-7
421	Indoxyl sulfate potassium salt	2642-37-7
422	3-(3-Hydroxyphenyl)propionic Acid	621-54-5
423	N-Acetyl-D-mannosamine	7772-94-3
424	2-Hydroxy Hippuric Acid	487-54-7
425	Sodium taurochenodeoxycholate	6009-98-9
426	3-Hydroxy-2-methyl-4-pyrone	118-71-8
427	Piperine	94-62-2
428	Homogentisic acid	451-13-8
429	L-Sorbose	87-79-6
430	(+)-4-Cholesten-3-one	601-57-0
431	1,2-Dipalmitoyl-sn-glycero-3-phosphoethanolamine	923-61-5
432	(+)-Catechin	154-23-4
433	Glycyl-L-leucine	869-19-2
434	Hexanoyl Glycine	24003-67-6
435	sn-glycero-3-Phosphocholine	28319-77-9
436	Guaiacol	90-05-1
437	Oleanolic acid	508-02-1
438	Docosanoic acid	112-85-6
439	cis-11-Eicosenoic acid	5561-99-9
440	Cholesteryl linoleate	604-33-1
441	3-Indolepropionic acid	830-96-6
442	N-Acetyl-L-ornithine	1572591
443	O-Phosphorylethanolamine	1071-23-4
444	N-Acetyl-L-tyrosine	537-55-3
445	Acetoin dimer	513-86-0
446	N-Acetyl-DL-serine	97-14-3
447	Methylguanidine HCl	21770-81-0
448	N-Acetyl-DL-Tryptophan	87-32-1
449	N-Methyl-L-glutamic acid	6753-62-4

450	4-Guanidinobutyric acid	463-00-3
451	N-Acetyl-L-leucine	1188-21-2
452	D-(-)-Quinic acid	77-95-2
453	L-Norvaline	6600-40-4
454	Pyrrole-2-carboxylic acid	634-97-9
455	2-Picolinic acid	98-98-6
456	Sodium alpha-ketoisocaproate	4502-00-5
457	D-Aspartic acid	1783-96-6
458	4-(Acetylamino)butanoic acid	3025-96-5
459	N-Acetyl-L-Proline	68-95-1
460	Carbocysteine	638-23-3
461	L-5-Hydroxytryptophan	895096
462	Cyclohexanecarboxylic Acid	98-89-5
463	Adenosine cyclophosphate	60-92-4
464	N-(4-Aminobutyl)acetamide hydrochloride	18233-70-0
465	4-Methylcatechol	452-86-8
466	Tryptamine	61-54-1
467	Methyl 3-indolyacetate	1912-33-0
468	Imidazole-4(5)-acetic Acid Hydrochloride	3251-69-2
469	Camphene	79-92-5
470	Glycyl-L-valine	1963-21-9
471	N-Acetyl-L-arginine dihydrate	210545-23-6
472	L-Leucyl-L-alanine Hydrate	7298-84-2
473	3-Ureidopropionic acid	462-88-4
474	Hypotaurine	300-84-5
475	Benzaldehyde	100-52-7
476	Caprylic acid	124-07-2
477	(S)-(−)-α-Methylbenzylamine	2627-86-3
478	Isobutyric acid	79-31-2
479	o-Cresol	95-48-7
480	(+)-Delta-Tocopherol	119-13-1
481	Hexanoic acid	142-62-1
482	2-Ketobutyric acid	600-18-0
483	Cyclohexanone	108-94-1
484	(+)-(S)-Carvone	2244-16-8
485	γ-caprolactone	695-06-7
486	2-Phenylethanol	60-12-8
487	Furfural	98-01-1

488	2-Methyl-3-Pentanone	565-69-5
489	4-Methyl-2-pentanone	108-10-1
490	α-Terpineol	98-55-5
491	2-Hexanone	591-78-6
492	Valeric acid	109-52-4
493	(S)-(+)-2-Methylbutyric acid	1730-91-2
494	3-Methylvaleric acid	105-43-1
495	Linoleic acid	60-33-3
496	Oleic acid	112-80-1
497	Heptanoic acid	111-14-8
498	Isovaleric acid	503-74-2
499	cis-4,7,10,13,16,19-Docosahexaenoic acid	6217-54-5
500	Palmitoleic Acid	373-49-9
501	Arachidonic Acid 250 mg in 1mL Ethanol	506-32-1
502	α-Linolenic Acid	463-40-1
503	Pyruvic Aldehyde sln aq 35-45%	78-98-8
504	Iso-Valeraldehyde	590-86-3
505	Nerol	106-25-2
506	(S)-(−)-Limonene	5989-54-8
507	1,2-Propanediol	57-55-6
508	(+)-α-Pinene	7785-70-8
509	(−)-α-Pinene	7785-26-4
510	Terpinen-4-ol	562-74-3
511	4-Methylvaleric acid	646-07-1
512	(±)-α-Tocopherol	10191-41-0
513	Glycerol	56-81-5
514	Spermidine	124-20-9
515	2-Undecanone	112-12-9
516	Methyl cyclohexanecarboxylate	4630-82-4
517	Dimethyl Trisulfide	3658-80-8
518	2-Methyl-1-propanol	78-83-1
519	2,3-Butanediol (mixture of isomers)	513-85-9
520	3-Methyl-1-butanol	123-51-3
521	N-(5-Aminopentyl)acetamide	32343-73-0
522	Pyruvic acid	127-17-3
523	m-Cresol	108-39-4
524	3-Methyl-2-oxobutanoic acid	759-05-7
525	β-Caryophyllene	87-44-5

526	cis-8,11,14-Eicosatrienoic Acid 1.5 mg EtOH sln	1783-84-2
527	Allyl Methyl Sulfide	10152-76-8
528	1-Butanol	71-36-3
529	cis-10-Nonadecenoic acid	73033-09-7
530	trans-2-Hexenal	6728-26-3
531	3-Hydroxyisovaleric acid	625-08-1
532	3-(Methylthio)propionic acid	646-01-5
533	(−)-β-Pinene	18172-67-3
534	Propionic acid	79-09-4
535	Benzyl alcohol	100-51-6
536	trans-Cinnamaldehyde	14371-10-9
537	Formamide	75-12-7
538	Pyrimidine	289-95-2
539	Styrene (stabilized with TBC)	100-42-5
540	1,4-Dichlorobenzene	106-46-7

Supplementary Figure S1. Another image corresponding to Figure 1C.

RPE/GFP-TSPAN4 cells were treated with cadaverine (50 μM) or putrescine (50 μM) for 24 hours before being imaged under a fluorescence microscope. Another image corresponding to Figure 1C.

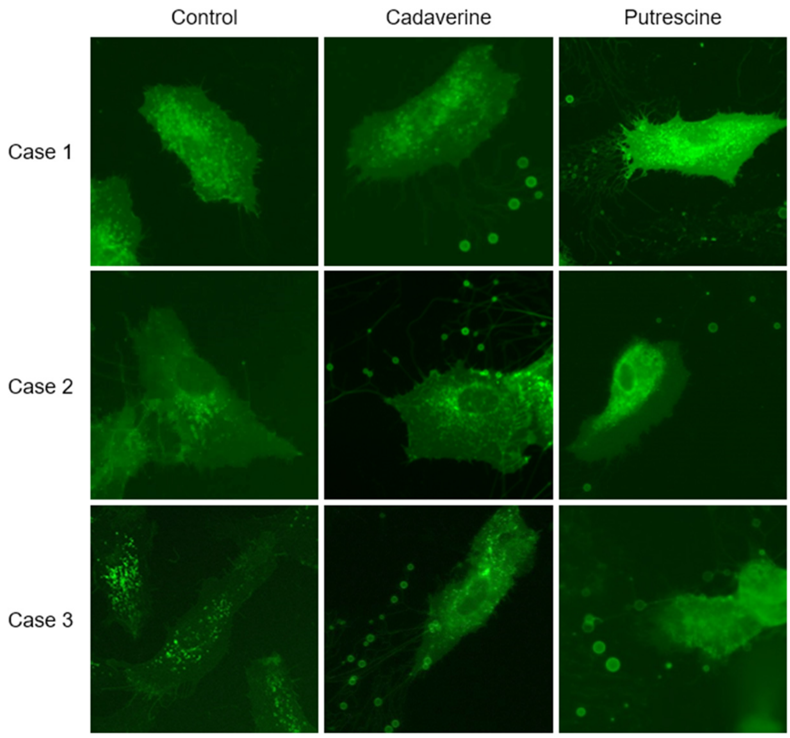

Supplementary Movie S1. Another image corresponding to Figure 1C.

To observe migrasome formation, GFP-TSPAN4-expressing RPE cells were treated with or without cadaverine and observed using Operetta CLS. Images were captured every 30 minutes for 24 hours.
